# 人淋巴瘤细胞Jurkat通过Fas/FasL途径抑制人肺癌细胞A549的免疫逃逸

**DOI:** 10.3779/j.issn.1009-3419.2010.07.05

**Published:** 2010-07-20

**Authors:** 红梅 王, 国强 张, 纪纲 戴, 家新 闵

**Affiliations:** 400037 重庆，第三军医大学附属新桥医院胸外科 Department of Thoracic Surgery, Xinqiao Hospital, Affiliated to the Third Military Medical University, Chongqing 400037, China

**Keywords:** 肺肿瘤, 凋亡, 免疫逃逸, Lung neoplasms, Apoptosis, Immune evasion

## Abstract

**背景与目的:**

当机体发生肿瘤时，肿瘤细胞可以多种方式逃避免疫系统的监控而分裂生长，这就是肿瘤的免疫逃逸。研究表明，肿瘤细胞自身凋亡减少以及机体免疫细胞凋亡增加是肿瘤免疫逃逸的重要机制。Fas/FasL系统是介导凋亡的重要分子体系，本研究旨在通过观察人淋巴瘤细胞株Jurkat诱导人肺癌细胞株A549凋亡过程中凋亡信号分子Fas、FasL及Caspase-8表达的改变，从而探讨Fas/FasL途径在肺癌细胞免疫逃逸中的作用。

**方法:**

人肺癌细胞株A549与人淋巴瘤细胞株Jurkat以不同比例分别进行共同培养，采用台盼蓝拒染法检测两种细胞存活率；流式细胞术（flow cytometry, FCM）检测两种细胞的凋亡率；Western blot技术检测A549细胞中Fas、FasL及Caspase-8蛋白表达水平。

**结果:**

随着Jurkat/A549细胞比例逐渐增大，A549细胞的凋亡率明显增加，Jurkat细胞凋亡率显著减少；同时A549细胞中Fas及Caspase-8蛋白的表达水平明显上调，而对FasL蛋白的表达水平无明显影响。

**结论:**

Jurkat细胞可能通过Fas/FasL途径介导了人肺癌细胞株A549的凋亡，从而抑制了A549细胞的免疫逃逸。

## Abstract (en)

**Background and objective:**

Tumor escape from the host immune system has been a major problem in immunotherapy of human malignancies.FasL/Fas-induced apoptosis plays an important role in various immunological processes.The aim of this study is to investigate the immune evasion in human lung carcinoma cell A549 induced by human lymphoma cell Jurkat via Fas/FasL pathway.

**Methods:**

Jurkat cells and A549 cells were co-cultured at different proportions.The apoptotic rates of A549 cells were determined by flow cytometry (FCM).Protein levels of Fas, FasL and Caspase-8 in A549 cells were detected by Western blot.

**Results:**

Survival rates of A549 cells gradually decreased and apoptotic rates of A549 cells were significantly enhanced along with ratio increasing between Jurkat and A549.Meanwhile, the protein levels of Fas and Caspase-8 gradually increased in A549 cells, and the protein levels of FasL had no significant difference in all groups.

**Conclusion:**

The Jurkat cells might decrease the survival rates of A549 cells and enhanced its apoptosis and immune evasion partly via Fas/FasL pathway.

肺癌是世界上最常见的恶性肿瘤之一，术后复发率和转移率均很高。近年来在手术治疗、放疗和化疗外，研究较多的是免疫治疗。对肺癌发病机制的研究发现，肺癌的发生发展与其自身的免疫逃逸机制密切相关。如果可通过某些途径遏制或阻断肺癌细胞的免疫逃逸，即能为肺癌的治疗提供新的机会。

当机体发生肿瘤时，肿瘤细胞可以多种方式逃避免疫系统的监控而分裂生长，这就是肿瘤的免疫逃逸^[1]^。研究^[2]^表明，肿瘤细胞自身凋亡减少以及机体免疫细胞凋亡增加是肿瘤免疫逃逸的重要机制。Fas/FasL系统是介导凋亡的重要分子体系^[3]^，当肺癌细胞与Jurkat细胞以一定比例共培养时，肺癌细胞可通过Fas/FasL途径诱导Jurkat细胞凋亡^[4]^。因此，为了探讨机体淋巴细胞是否可以通过Fas/FasL途径抑制肺癌细胞的免疫逃逸，本研究以人肺癌细胞株A549和人T细胞淋巴瘤细胞株Jurkat为研究对象，观察不同细胞比例共培养下对Jurkat细胞生长的影响；同时观察不同比例共培养对A549和Jurkat细胞各自凋亡率的影响；最后检测A549细胞中凋亡相关信号分子Fas、FasL及Caspase-8蛋白表达情况，探讨Fas/FasL通路在Jurkat细胞诱导A549细胞凋亡及抑制肺癌细胞免疫逃逸中的作用，为临床研究以Fas/FasL为靶点治疗肺癌提供一定的实验依据。

## 1 材料与方法

### 1.1 材料

人肺癌细胞株A549由第三军医大学新桥医院呼吸研究所提供，人淋巴瘤细胞株Jurkat（ATCC TIB-152）购自上海麦莎生物科技有限公司。Annexin V-FITC凋亡检测试剂盒购自杭州联科生物技术有限公司（货号：K101-100）。兔抗人Fas、FasL、Caspase-8单克隆抗体由美国Santa Cruz公司提供。

### 1.2 方法

#### 1.2.1 细胞培养

A549细胞及Jurkat细胞在5%CO_2_、37 ℃条件下用含10%优质胎牛血清的RPMI-1640培养基进行复苏、传代。将A549细胞以2×10^5^/mL密度接种至培养瓶内，待细胞长满至85%左右时，吸去培养基；用新鲜培养基将对数生长期Jurkat细胞制成细胞悬液，按1:1、10:1、20:1、50:1（A549/Jurkat）加入A549细胞培养瓶中共培养，同时设置对照组A549及Jurkat细胞，6 h后分别收集两种细胞。分别用3%台盼蓝染色，计数存活细胞数量。

#### 1.2.2 流式细胞术检测A549及Jurkat细胞的凋亡

分别收集各组中两种细胞，用PBS洗涤2次，加入150 μL Binding Buffer和FITC标记的Annexin-V 5 μL，室温避光15 min，再加入PI 5 μL，避光反应5 min后，加入200 μL Binding Buffer，立即用流式细胞仪检测A549与Jurkat细胞的凋亡率，同时以不加AnnexinV-FITC及PI的一管作为阴性对照。以上实验每组同设至少3个样本，该实验至少重复3次。

#### 1.2.3 各组A549细胞中Fas、FasL、Caspase-8蛋白水平检测

分别收集实验中各组A549细胞，提取细胞总蛋白后用考马斯亮蓝试剂盒检测蛋白浓度，调整样本浓度基本一致后进行蛋白变性处理，保存于-20 ℃。分别将蛋白样本进行凝胶电泳（SDS-PAGE）后，半干法转膜至PVDF膜上（15 V, 20 min），5%脱脂奶粉封闭2 h，随后分别用兔抗人Fas（43 kDa）、FasL（40 kDa）、Caspase-8单克隆抗体和GAPDH单克隆抗体4 ℃孵育过夜。TBST洗膜后再加入辣根过氧化物（HRP）标记羊抗兔IgG（1:1 000），4 ℃孵育2 h。洗膜后用化学发光底物显影，凝胶图像处理系统软件（Brad, USA）分析目的条带的灰度值。结果判断：以目的蛋白的灰度值与GAPDH的灰度值的比值表示各目的蛋白相对表达水平。

### 1.3 统计学处理

采用SPSS 13.0统计软件进行数据分析，所有计量资料以Mean±SD表示。两组间比较采用*t*检验，组间比较采用单因素方差分析（*ANOVA*）。以*P* < 0.05为差异有统计学意义。

## 2 结果

### 2.1 A549与Jurkat细胞以不同比例共培养时对Jurkat细胞生长的影响

人肺癌细胞与Jurkat细胞共培养6 h后，台盼蓝染色细胞计数发现，随着A549/Jurkat比例逐渐增大，Jurkat细胞生长明显受到抑制，各组间存在着明显差异（*P* < 0.05, *P* < 0.01），且比例越大抑制率越高（图 1）。

**1:**
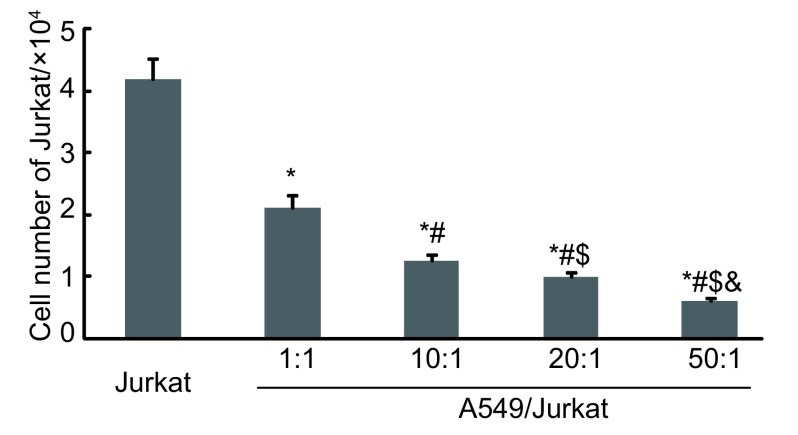
台盼蓝染色检测各组中Jurkat细胞存活率。A549细胞与Jurkat细胞按0、1:1、10:1、20:1、50:1进行共培养，随着A549细胞数增加，Jurkat细胞存活率逐渐降低。^*^P < 0.01（*vs* 0）；^#^*P* < 0.01（*vs* 1:1）；^$^*P* < 0.01（*vs* 10:1）； & *P* < 0.01（*vs* 20:1）。 Jurkat cells survival rates in all groups detected by trypan blue dyeing.A549 cells and Jurkat cells co-cultured in proportion to 0, 1:1, 10:1, 20:1, 50:1.Jurkat cells survival rates reduced along with A549 cells increased.^*^P < 0.01 (*vs* 0);^#^*P* < 0.01 (*vs* 1:1);^$^*P* < 0.01 (*vs* 10:1); ^&^*P* < 0.01 (*vs* 20:1).

### 2.2 A549与Jurkat细胞以不同比例共培养时对A549及Jurkat细胞凋亡的影响

随着A549/Jurkat比例逐渐增大，Jurkat细胞凋亡率明显增加，而A549细胞凋亡率明显降低，各组间存在着显著差异，且差异具有统计学意义（*P* < 0.05, *P* < 0.01），提示增加的A549细胞促进了Jurkat细胞的凋亡，同时A549细胞免疫逃逸能力增强（图 2）。

**2:**
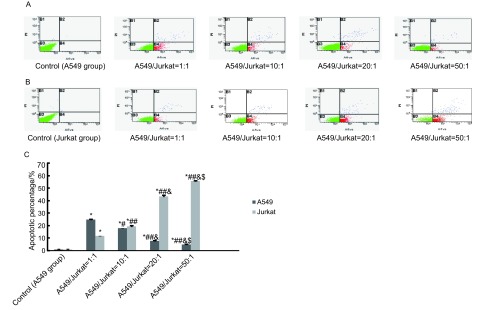
流式细胞术检测各组中A549、Jurkat细胞凋亡率。A：流式细胞术检测各组中A549细胞凋亡率；B：流式细胞术检测各组中Jurkat细胞凋亡率；C：A549与Jurkat细胞以不同比例混合时A549、Jurkat细胞凋亡率。随着Jurkat细胞比例减少及A549细胞比例的增加，A549细胞凋亡率逐渐减少，Jurkat细胞凋亡率逐渐增加。随着Jurkat细胞比例减少及A549细胞比例的增加，A549细胞凋亡率逐渐减少，Jurkat细胞凋亡率逐渐增加。 Apoptotic rates of A549 cells and Jurkat cells in all groups detected by FCM.A: Apoptotic rates of A549 cells in all groups detected by FCM; B: Apoptotic rates of Jurkat cells in all groups detected by FCM; C: Apoptotic rates of Jurkat cells and A549 cells in all groups.Apoptotic rates of Jurkat cells reduced and apoptotic rates of A549 cells increased along with A549/Jurkat gradually increasing.^*^P < 0.01 (*vs* 0); ^#^*P* < 0.05, ^##^*P* < 0.01(*vs* 1:1); ^&^*P* < 0.01 (*vs* 10:1); ^$^*P* < 0.01 (*vs* 20:1).

### 2.3 A549与Jurkat细胞以不同比例共培养时对A549细胞中凋亡相关分子Fas、FasL及Caspase-8蛋白表达的影响

随着A549/Jurkat比例逐渐减小，A549细胞中Fas、Caspase-8蛋白水平明显上调，而FasL蛋白水平未发生明显改变，提示增加的Jurkat细胞有效地上调了A549细胞中Fas、Caspase-8蛋白水平，从而促进其凋亡（图 3，表 1）。

**3:**
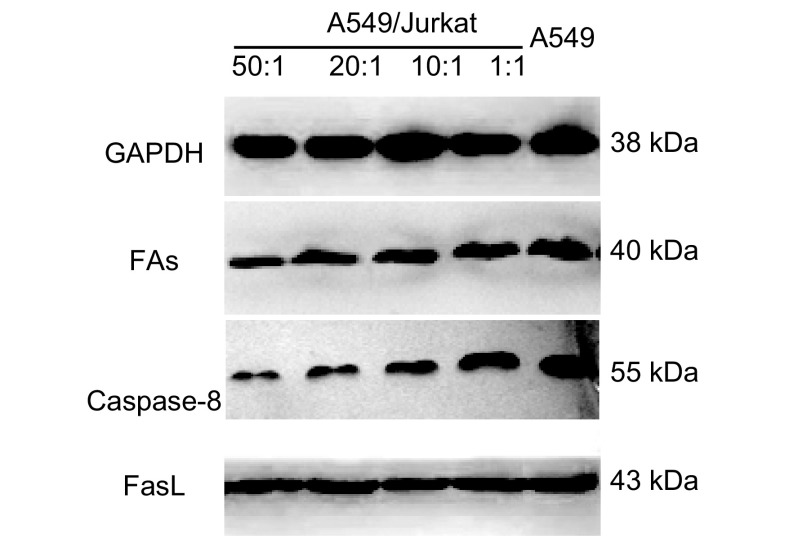
各组A549细胞中FasL、Fas及Caspase-8蛋白检测 Expressions of FasL, Fas and Caspase-8 in A549 cells

**1:** A549细胞中Fas、FasL以及Caspase-8蛋白的表达（Mean±SD） Protein expressions of Fas, FasL and Caspase-8 in A549 cells (Mean±SD)

Group	Control (A549 group)	A549/Jurkat=50:1	A549/Jurkat=20:1	A549/Jurkat=10:1	A549/Jurkat=1:1
Fas	0.443 3±0.015 3	0.440 0±0.010 0^**^	0.496 7±0.005 8^*^	0.560 0±0.010 0^*^	0.603 3±0.015 3^*^
FasL	0.613 3±0.020 8	0.636 7±0.011 6^***^	0.623 3±0.015 3^***^	0.620 0±0.017 3^***^	0.633 3±0.015 3^***^
Caspase-8	0.446 7±0.011 6	0.453 3±0.005 8^**^	0.506 7±0.015 3^*^	0.553 3±0.011 6^*^	0.603 3±0.015 8^*^
^*^: *P* < 0.05, ^***^: *P* > 0.05, compared among experimental groups; ^**^: *P* > 0.05, compared with the control group. Experimental groups were 50:1, 20:1, 10:1, 1:1 (A549/Jurkat); the control group was only A549 cells.					

## 3 讨论

Fas，又名CD95或APO-1，属于肿瘤坏死因子受体（tumor necrosis factor receptor, TNFR）和神经生长因子受体（nerve growth factor receptor, NGFR）家族，是相对分子量为4 500的Ⅰ型膜蛋白。机体中许多组织细胞可表达或经激活诱导表达Fas，以免疫系统的表达最丰富。Fas配体（FasL）是一种相对分子量约4 000的三聚体Ⅱ型跨膜糖蛋白，不仅在免疫豁免器官如睾丸中表达，还可在多数肿瘤细胞表达。FasL与Fas结合后，诱导Fas形成三聚体，并将凋亡信号传递给Caspase-8。Caspase-8是一种蛋白酶，是半胱氨酸蛋白酶超家族中的一员。在Fas引发的凋亡过程中，一系列Caspase级联式地活化，其中Caspase-8的活化是其第一步反应。活化后的Caspase-8会引发下游Caspase活化，进而诱导细胞凋亡^[5]^。研究^[6]^表明肺癌细胞通过高表达FasL，诱导机体表达Fas的淋巴细胞凋亡，从而逃避机体免疫清除，而Fas、Caspase-8同时也参与了这一过程。

通过本研究发现，增加共培养体系中Jurkat细胞数量有利于提高肺癌细胞A549中Fas、Caspase-8的表达水平，而对FasL的表达无明显影响。我们推测Jurkat细胞数量的增加有助于抑制肺癌细胞A549的免疫逃逸，从而促进肺癌细胞的凋亡。本结果与向青等^[4]^研究基本一致，同时我们也注意到，当Jurkat细胞数量增加时，肺癌细胞A549的Fas反击效应并未显现出来，推测可能与肿瘤浸润过程中*FasL*基因发生突变、FasL表达水平无明显改变密切相关。而增加的Fas、Caspase-8则明显增强了Fas/FasL系统的清除能力，从而有效地诱导了肺癌细胞的凋亡。

已有研究^[7]^表明Caspase-8在神经母细胞瘤、肝癌、宫颈癌等疾病的发生发展中起重要作用。对食管癌的研究^[8]^已经证实，从食管正常黏膜组织到不典型增生和食管癌组织，Caspase-8蛋白及RNA阳性表达率呈逐渐下降趋势，而在高、中分化鳞癌中的表达率高于低分化鳞癌。也有研究^[9, 10]^表明*Caspase-8*基因经常突变或沉默，从而引起肿瘤细胞的化疗抵抗，如肝癌和肾癌等。本研究结果显示Caspase-8参与了A549细胞凋亡的发生，随着凋亡的增加，A549细胞表达Caspase-8也增加。由此我们可以设想，通过基因技术改变机体免疫细胞或肿瘤细胞表达Caspase-8，从而遏制肿瘤生长、转移，达到治疗肿瘤的目的。

当肿瘤细胞受到Fas阳性的T细胞攻击时，肿瘤细胞可通过减少Fas的表达逃避T淋巴细胞对其的攻击作用^[2, 11]^；同时，肿瘤细胞可通过高表达FasL对T淋巴细胞展开反攻击^[12]^；并且，一些肿瘤细胞虽然表达Fas，但其却对抗T淋巴细胞性FasL介导的凋亡效应。Fas的突变同样可导致凋亡信号传导的异常，其最终效应是诱导宿主对抗肿瘤细胞的死亡，最终产生免疫抑制效应。本研究提示，肺癌的发生与发展过程与Fas/FasL系统异常所致的机体免疫清除功能部分失活密切相关。同时还可以推测，随着肺癌病程的发展，机体免疫细胞中某些基因可能发生了突变，导致Fas、FasL、Caspase-8的表达发生改变、细胞清除肿瘤细胞的能力降低以及免疫防御作用减弱，从而有利于肿瘤的扩散和转移。

事实上，Fas/FasL通路只是肺癌细胞众多免疫逃逸机制之一^[13]^，其在肺癌细胞免疫逃逸中的地位及与其它相关机制的相互作用还有待进一步阐明。我们也期待肺癌的免疫治疗能有新的进展。
